# *Ganoderma lucidum* Polysaccharides Ameliorate Acetaminophen-Induced Acute Liver Injury by Inhibiting Oxidative Stress and Apoptosis along the Nrf2 Pathway

**DOI:** 10.3390/nu16121859

**Published:** 2024-06-13

**Authors:** Nan Zhang, Zhongming Han, Rui Zhang, Linling Liu, Yanliang Gao, Jintao Li, Meixia Yan

**Affiliations:** 1 Institute of Special Animal and Plant Sciences of Chinese Academy of Agricultural Sciences, Changchun 130112, China; yyyzxr@163.com (N.Z.); liulinling@caas.cn (L.L.); gaoyanliang@caas.cn (Y.G.); lijintao@caas.cn (J.L.); 2 College of Traditional Chinese Medicinal Materials, Jilin Agricultural University, Changchun 130118, China; hanzm2008@126.com (Z.H.); zr99101@163.com (R.Z.); 3 Jilin Provincial Key Laboratory of Traditional Chinese Medicinal Materials Cultivation and Propagation, Changchun 130112, China

**Keywords:** *Ganoderma lucidum* polysaccharides, acute liver injury, oxidative stress, Nrf2 signaling pathway

## Abstract

The excessive employment of acetaminophen (APAP) is capable of generating oxidative stress and apoptosis, which ultimately result in acute liver injury (ALI). *Ganoderma lucidum* polysaccharides (GLPs) exhibit hepatoprotective activity, yet the protective impact and potential mechanism of GLPs in relation to APAP-induced ALI remain ambiguous. The intention of this research was to scrutinize the effect of GLPs on APAP-induced ALI and to shed light on their potential mechanism. The results demonstrated that GLPs were capable of notably alleviating the oxidative stress triggered by APAP, as shown through a significant drop in the liver index, the activities of serum ALT and AST, and the amounts of ROS and MDA in liver tissue, along with an increase in the levels of SOD, GSH, and GSH-Px. Within these, the hepatoprotective activity at the high dose was the most conspicuous, and its therapeutic efficacy surpassed that of the positive drug (bifendate). The results of histopathological staining (HE) and apoptosis staining (TUNEL) indicated that GLPs could remarkably inhibit the necrosis of hepatocytes, the permeation of inflammatory cells, and the occurrence of apoptosis induced by APAP. Moreover, Western blot analysis manifested that GLPs enhanced the manifestation of Nrf2 and its subsequent HO-1, GCLC, and NQO1 proteins within the Nrf2 pathway. The results of qPCR also indicated that GLPs augmented the expression of antioxidant genes Nrf2, HO-1, GCLC, and NQO1. The results reveal that GLPs are able to set off the Nrf2 signaling path and attenuate ALI-related oxidative stress and apoptosis, which is a potential natural medicine for the therapy of APAP-induced liver injury.

## 1. Introduction

Acute liver injury (ALI) caused by drugs, alcohol, viruses, diet, or other elements has turned into a crucial reason for acute liver failure (ALF) worldwide [1]. Within this context, drug-induced liver injury (DILI) stands out as one of the most frequent and severe types of negative drug reactions [2]. It is approximated that the annual incidence of DILI in the general population in mainland China amounts to 23.8 cases per 100,000 individuals, whereas in Western countries, it ranges from 1 to 20 cases per 100,000 people [3]. Acetaminophen (APAP), which is a commonly employed antipyretic and analgesic medicine in clinical settings, has a notable therapeutic effect at a safe dose. When used excessively, it can cause ALI, and in severe cases, ALF can occur [4]. The incidence of DILI caused by APAP has been increasing progressively over the years, and it has become the primary factor contributing to ALF in Western countries [5]. Up to now, N-acetylcysteine (NAC) has been the only antidote that has been sanctioned for the clinical treatment of APAP-induced liver injury [6]. However, since NAC can only bring its greatest impact within 8 h after APAP poisoning, and the treatment window is narrow along with a host of adverse reactions [7], it is significant to seek out more efficacious and less side-effect-laden drugs.

After the therapeutic dose of APAP enters the liver, approximately 90% of APAP is transformed into non-poisonous compounds and excreted from the body under the influence of phase II metabolic reaction binding enzymes UDP glucuronyltransferase (UGT) and sulfotransferase (SULT). The remaining 10% of APAP is oxidized by the cytochrome P450 enzyme to generate n-acetyl-p-benzoquinone imine (NAPQI) cytotoxic compounds [8]. Glutathione (GSH) in the liver combines with NAPQI and is discharged into the bile to avert tissue damage. In the event of excessive APAP, the glucuronic acid or sulfation pathway in phase II metabolism becomes saturated, and more NAPQI is produced via the CYP2E1 pathway, with GSH gradually being depleted. The remaining NAPQI is combined with intracellular proteins, DNA, and unsaturated lipids, giving rise to oxidative stress and mitochondrial dysfunction, ultimately culminating in the demise of hepatocytes [9]. Furthermore, nuclear factor erythroid 2-related factor 2 (Nrf2), which is a factor that can enhance the exhibition of various detoxification and antioxidant-related genes in numerous antioxidant signaling pathways, typically binds to the inhibitory factor Keap1 (kelch-like ECH-associated protein 1) in the cytoplasm [10]. During antioxidant defense, it separates from Keap1 and is released into the nucleus, triggering the activation of the transcription of downstream genes such as heme oxygenase-1 (HO-1), the glutamic subunit of glutamate cysteine ligase (GCLC), the glutamate–cysteine ligase modifier subunit (GCLM), human quinone oxidoreductase 1 (NQO1), and other antioxidant genes, thereby regulating the expression of various antioxidant enzymes, alleviating oxidative stress [11], inhibiting the increase in ROS levels, and reducing the expression of inflammatory factors; consequently, apoptosis is suppressed. Therefore, the NRF2/KEAP1 pathway is a key chemotherapy target for many cancer and non-cancer diseases [12].

*Ganoderma lucidum* is a large kind of conventional edible and medicinal fungus that has a history of over 2000 years. It has significant medicinal value. Polysaccharides, as one of the crucial active constituents of *Ganoderma lucidum*, indeed mainly comprise glucose, arabinose, galactose, xylose, and mannose [13]. The structural characterization and pharmacological activities of *Ganoderma lucidum* polysaccharides (GLPs) have been the subjects of the most in-depth investigations [14]. Some studies have demonstrated that GLPs are capable of reducing liver toxicity caused by alcohol [15], carbon tetrachloride [16], BCG [17], and so on. Previous studies conducted by the team revealed that *Ganoderma lucidum* spore polysaccharide powder is capable of inhibiting APAP-induced apoptosis, enhancing antioxidant capabilities, and alleviating APAP-induced liver injury, suggesting that it might be one of the components that play a safeguarding function in APAP-induced liver injury [18]. However, it remains to be investigated whether polysaccharides from *Ganoderma lucidum* fruiting bodies possess hepatoprotective capacity in resistance to APAP-induced liver lesions.

Therefore, polysaccharides from the fruiting body of *Ganoderma lucidum* were extracted, and their molecular weight and monosaccharide composition were determined. The intent of this study was to investigate the hepatoprotective potential and mechanism of GLPs. This was accomplished by selecting biphenyl diester as a positive control drug [19] via the mouse model of APAP liver injury, with a focus on the impact of activating the Nrf2 pathway on oxidative stress and cell apoptosis. This provides a theoretical foundation for the further development of *Ganoderma lucidum* in the prevention and treatment of APAP liver injury, thereby laying the groundwork for future advancements.

## 2. Materials and Methods

### 2.1. Materials and Reagents

*Ganoderma lucidum* was provided by the Institute of Special Animal and Plant Sciences CAAS (Changchun, China). The chemical reagents encompassing sodium nitrate (item number 7631-99-4), dimethyl sulfoxide (item number 67-68-5), trifluoroacetic acid (item number 76-05-1), and sodium acetate (item number 127-09-3) were procured from Merck Bioscience (Darmstadt, Germany). The monosaccharide standard was provided by Sigma-Aldrich (St. Louis, MO, USA). The test kits for aspartate aminotransferase (AST/GOT) (item number C010-2-1), alanine aminotransferase (ALT/GPT) (item number C009-2-1), superoxide dismutase (SOD) (item number A001-3), glutathione peroxidase (GSH-Px) (item number A005-1), trace reduced glutathione (GSH) (item number A006-2-1), and trace malondialdehyde (MDA) (item number A003-2) were all supplied by the Nanjing Jiancheng Bioengineering Institute (Nanjing, China). The reactive oxygen species (ROS) ELISA kit (item number YX-181519) was bought from Shanghai Enzyme-linked Biotechnology Co., Ltd. (Shanghai, China). The antibodies including rabbit anti-mouse Nrf2, HO-1, GCLC, NQO1, and β-actin were purchased from ABclonal Technology Co., Ltd. (Wuhan, China), and goat anti-rabbit IgG HRP was purchased from Beyotime Biotech, Inc. (Shanghai, China). The primer synthesis for the five genes, namely Nrf2, HO-1, GCLC, NQO1, and β-actin, was performed by Sangon Biotech Co., Ltd. (Shanghai, China). The histopathology HE staining kit (item number C0105S) and the apoptosis TUNEL staining kit (item number C1091) were obtained from Beyotime Biotech, Inc. (Shanghai, China). Other reagents used in this trial were procured from local chemical suppliers.

### 2.2. Preparation of GLPs

**Figure 1:**
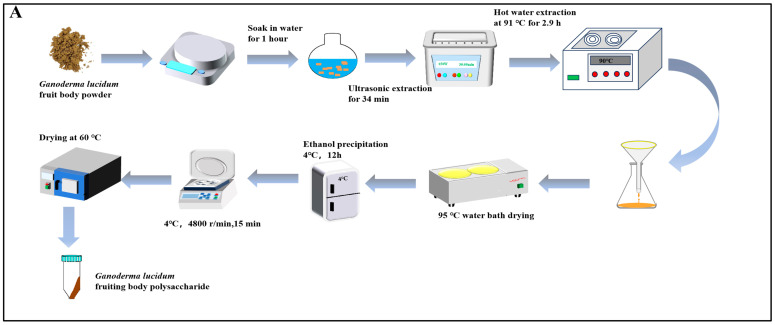

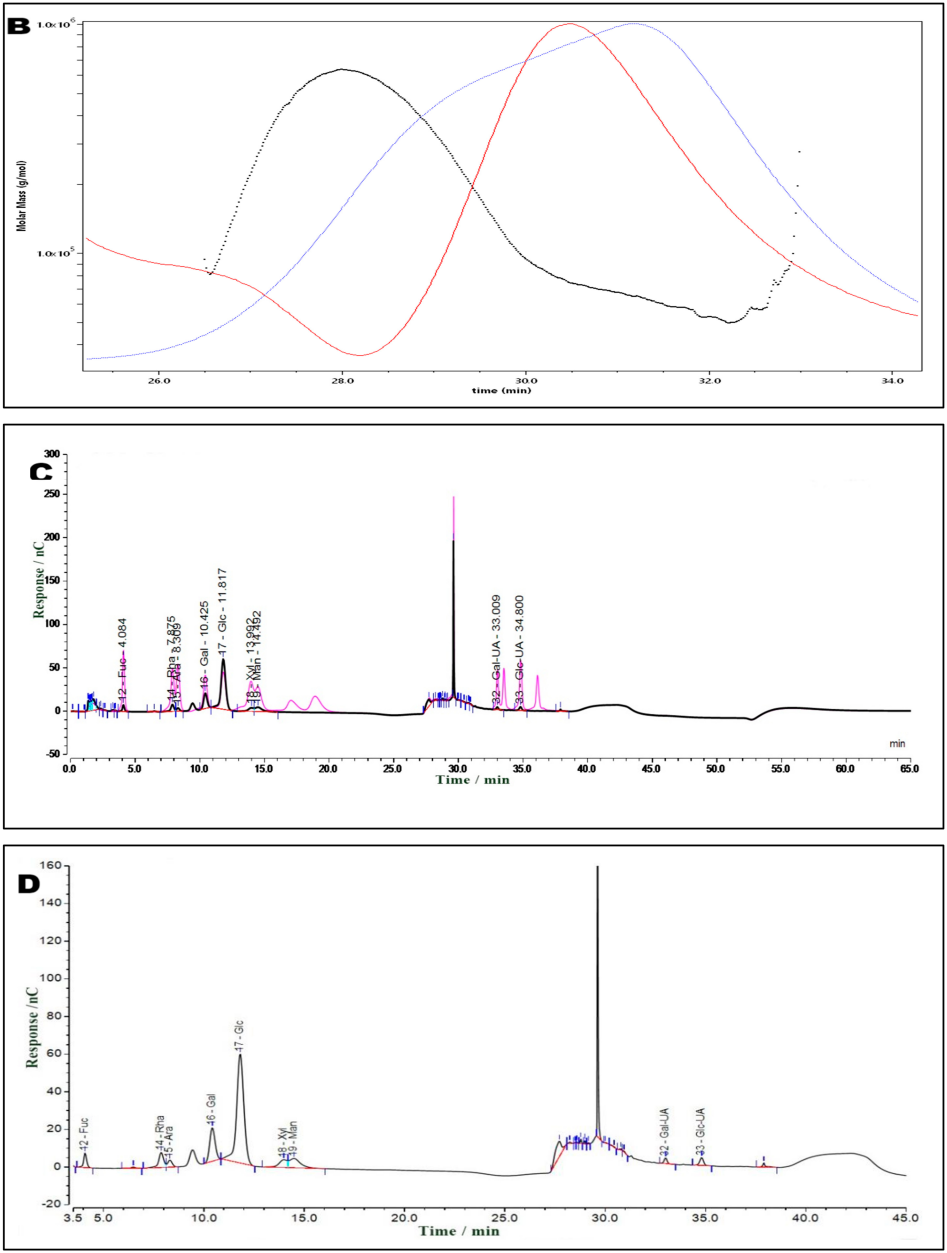
(**A**) Flowchart of ultrasonic-assisted hot water extraction of GLPs; (**B**) polysaccharides’ molecular weight (The red line represents the multi angle laser light scattering signal, the blue line represents the differential signal, and the black line is the molecular weight fitted by the two signals); (**C**) standard; (**D**) monosaccharide composition.

As presented in Figure 1A, the crude polysaccharides of *Ganoderma lucidum* were extracted by ultrasonic-assisted hot water extraction. The dried crude polysaccharides were thoroughly dissolved in distilled water, and then it was centrifuged at a speed of 7500 r/min for 10 min, after which the supernatant was taken away. The protein was eliminated using the Sevage method (n-butanol–chloroform = 1:4), and this process was repeated several times. The crude polysaccharide solution after the removal of protein was transferred into a dialysis bag with a cut-off flow of 3500 Da. The small molecular impurities in the polysaccharide were removed through dialysis with distilled water for 48 h. After the dialysis, the polysaccharide solution was freeze-dried to obtain the refined crude GLP.

### 2.3. Determination of Polysaccharide Content

The total sugar content was probed by means of the phenol–sulfuric acid method with glucose as the benchmark [20].

### 2.4. Absolute Molecular Weight Determination

The molecular weight of GLPs was ascertained via the gel chromatography differential multi-angle laser light scattering system [21]. The liquid phase system was U3000 (Thermo, Waltham, MA, USA); the differential detector was Optilab T-rEX (Wyatt Technology, Santa Barbara, CA, USA); the laser light scattering detector was DAWN HELEOS II (Wyatt Technology, CA, USA); and the gel columns, ohpak SB-805 HQ (300 × 8 mm, Showa Electric, Tokyo Metropolitan Area, Japan) and ohpak SB-803 HQ (300 × 8 mm, Showa Electric, Tokyo Metropolitan Area, Japan), were connected in series. The mobile phase consisted of 0.02% NaN_3_ and 0.1 M NaNO_3_, with gradient elution applied. The flow rate was 0.6 mL/min, the column temperature was 45 °C, and the chromatographic data were processed using the software Astra 6.1.

### 2.5. Monosaccharide Composition Determination

In accordance with the approach of previous studies [22], the composition of monosaccharides was determined by the Thermo ICS5000 ion chromatography system (ICS5000, Thermo Fisher Scientific, Waltham, MA, USA)—Dionex ™ Carbopac ™ PA20 (150 × 3.0 mm, 10 μm) through high-performance liquid chromatography (HPLC). Water (H_2_O) was used as mobile phase A; 0.1 M NaOH was used as mobile phase B; 0.1 M NaOH and 0.2 M NaAc were used as mobile phase C; and the flow rate was 0.5 mL/min. The standards of monosaccharides included glucose, galactose, rhamnose, mannose, xylose, fucose, galacturonic acid, glucuronic acid, and arabinose.

### 2.6. Animal Experiments

#### 2.6.1. Animals

A total of sixty clean-grade male ICR mice, with a body weight of (20 ± 2) g, were supplied by Liaoning Changsheng Biotechnology Co., Ltd. (Benxi City, Liaoning Province, China). The mice were kept in a specific pathogen-free environment, where the temperature ranged from 22 to 25 °C, the relative humidity was between 40% and 70%, and there was a 12 h light/dark cycle. Standard rodent food and water were provided. The animal experiment was authorized by the Laboratory Animal Management and Welfare Ethics Committee of the Institute of Specialty of the Chinese Academy of Agricultural Sciences.

#### 2.6.2. Experimental Design

**Figure 2:**
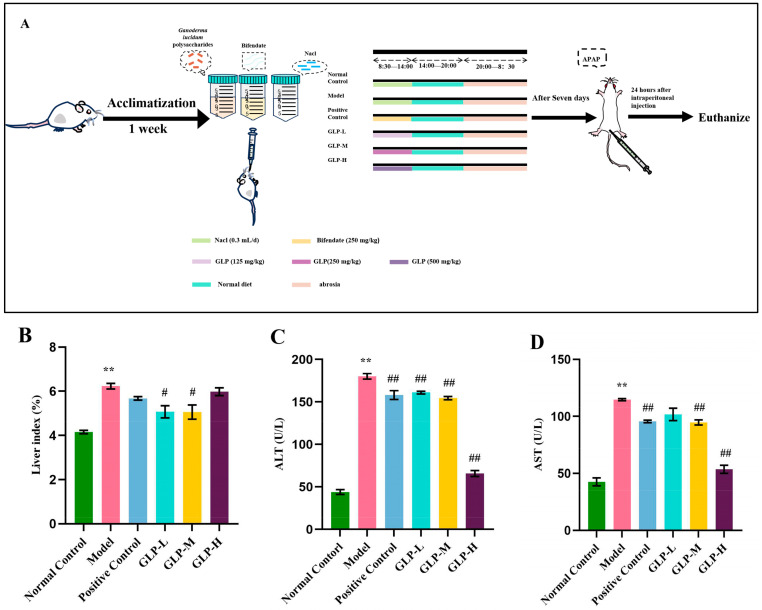
Effect of GLPs on APAP-induced liver injury in mice: (**A**) experimental design; (**B**) liver index; (**C**) ALT activity; (**D**) AST activity *(n* = 6). The results are expressed as mean ± standard error; compared with the normal group, ** *p* < 0.01; compared with the model, # *p* < 0.05, ## *p* < 0.01. Normal control: blank control group, model: model group, positive control: positive group, GLP-L: *Ganoderma lucidum* polysaccharide low-dose group, GLP-M: *Ganoderma lucidum* polysaccharide medium-dose group, GLP-H: *Ganoderma lucidum* polysaccharide high-dose group.

As demonstrated in Figure 2A, after 7 days of acclimation feeding, 60 mice were randomly separated into 6 groups, which consisted of a blank control group, a model group, a low-dose GLP group (125 mg/kg), a medium-dose GLP group (250 mg/kg), a high-dose GLP group (500 mg/kg), and a positive control group (diphenyl diester 300 mg/kg), with 10 mice in each group. The mice in the blank control group and the model group were given 0.3 mL of normal saline by gavage at regular intervals every day; the mice in the low-dose, medium-dose, and high-dose polysaccharide groups and the positive control group were gavaged with the corresponding dose of solution at regular intervals every day. Throughout the entire experiment, normal eating and drinking were maintained, and every day at 20:00, food was fasted. On the next day, at a fixed time and in a fixed space, the samples were administered by abdominal gavage, and then feed was provided. The drug was administered by gavage for 7 days. Except for the blank group, the other groups received an intraperitoneal injection of APAP at 250 mg/kg 1 h after the last administration (in accordance with the previous experiment). After modeling, fasting and drinking were prohibited. After 24 h, blood was collected from the posterior orbital venous plexus. After standing at room temperature for 1 h, the serum was centrifuged at 3500 r/min for 15 min. The serum was taken and refrigerated at 4 °C. Liver tissues were also obtained for histopathological inspection and protein extraction.

#### 2.6.3. Liver Histological Analysis

The liver tissue samples were fixed in 10% neutral buffered formaldehyde solution for 48 h. Afterward, the liver samples were fixed and embedded in paraffin blocks. The tissue specimens were sliced into 4 μm thick sections. These sections were dewaxed with xylene and hydrated with ethanol at different gradients in sequence. After that, they were tinted with hematoxylin and eosin and finally scanned under the Olympus VS200 (Olympus Corporation, Tokyo, Japan)system to observe the pathological changes.

#### 2.6.4. TUNEL Cell Apoptosis Detection

In line with the directions of the TUNEL cell apoptosis detection kit (chromogenic method), TUNEL detection was conducted on the formalin-fixed and paraffin-embedded sections. The peroxidase activity was revealed by diaminobenzidine (DAB), and the hematoxylin staining solution was employed as a re-staining agent for the slide. Subsequently, the positive cells were observed under the light microscope.

#### 2.6.5. Serum and Liver Biochemical Assays

Serum and liver underwent biochemical analyses were performed using various test kits. The serum activities of ALT and AST were determined employing the alanine aminotransferase (ALT/GPT) test kit and the aspartate aminotransferase (AST/GOT) test kit for evaluating liver injury. The liver tissue was mixed with normal saline in a 1:9 mass ratio, homogenized, and then centrifuged at 3000 r/min at 4 °C for 10 min, yielding the supernatant. The superoxide dismutase (SOD) assay kit, glutathione peroxidase (GSH-Px) test kit, trace reduced glutathione (GSH) assay kit, and trace malondialdehyde (MDA) test kit were utilized for determining the activities of SOD, GSH-Px, GSH, and MDA. The OD value of the sample was measured using a microplate reader (BioTek, American Berton Instruments Co., Ltd., Winooski, VT, USA).

#### 2.6.6. Enzyme-Linked Immunosorbent Assay

The mouse liver that had been stored in the refrigerator at −80 °C was taken out, and an appropriate amount of physiological saline was added for homogenization. Then, it was centrifuged at 3000 r/min for 10 min at 4 °C, and the supernatant was obtained. In accordance with the manufacturer’s instructions, the ROS levels were determined by using the mouse reactive oxygen species (ROS) ELISA Kit (Shanghai Enzyme-linked Biotechnology Co., Ltd., Shanghai, China).

#### 2.6.7. Western Blot Analysis

Mouse liver specimens were disrupted on ice by using the Ripa buffer with protease inhibitor for 30 min. After centrifugation at 20,000× *g* (4 °C) for 15 min, the protein content of the sample was determined using the BCA method. Proteins were separated using SDS polyacrylamide gel (50 μg per lane) and then transferred onto the methanol-activated PVDF membrane. The film was blocked with 5% skimmed milk at ambient temperature for 2 h, after which it was immersed in a properly diluted antibody solution and incubated overnight at 4 °C, with the following antibodies: anti-Nrf2 (ABclonal), anti-HO-1 (ABclonal), anti-GCLC (ABclonal), anti-NQO1 (ABclonal), and anti-β-actin (ABclonal). The antibody dilution ratio is shown in Table 1. Subsequently, the membrane was incubated together with horseradish peroxidase-conjugated anti-rabbit (Beyotime Biotech) secondary antibody at room temperature for 2 h. Enhanced chemiluminescence was carried out using the Takara ECL Western blot kit as per the manufacturer’s instructions (item number T7101A, Takara, Osaka City, Japan).

#### 2.6.8. Quantitative Real-Time Polymerase Chain Reaction (qRT-PCR)

In accordance with the instructions provided by the kit, total RNA was extracted from the mouse liver using the TRNzol universal total RNA Extraction Reagent (Tiangen, Beijing, China). The cDNA was synthesized using 2 × rapid Taq Master Mix (Vazyme, Nanjing City, China). SYBR^®^ Green (Vazyme) was utilized to perform qRT-PCR on the Quantum Studio™ real-time PCR detection system (Thermo Fisher). The expression levels of Nrf2, HO-1, GCLC, and NQO1 were quantified, with β-actin serving as an internal control. The relative expression degree of the target gene was computed by means of the 2^−ΔCT^ approach. The primers are presented in Table 2.

### 2.7. Statistical Analysis

All statistical analyses were performed by utilizing SPSS version 26.0 (IBM, Amonk, NY, USA), and the results are reported as the mean ± SEM. One-way analysis of variance (ANOVA) was utilized to assess the discrepancy between the two groups, considering *p* < 0.05 as statistically significant. GraphPad Prism 9 (GraphPad Software, La Jolla, CA, USA) was used for Tukey multiple-comparison analysis, where *p* < 0.05 indicates a significant difference, and *p* < 0.01 indicates an extremely remarkable difference.

## 3. Results

### 3.1. Physical and Chemical Properties

The crude polysaccharide powder after extraction and purification was brown, and the standard curve established with glucose as the reference substance was y = 0.0039x + 0.0384 (R^2^ = 0.9982). The total sugar content of the purified crude polysaccharides was 89.85 ± 4.02%. The results showed that the purity of polysaccharides in the crude polysaccharides was high. The main elution time range of GLPs was from 29.0 to 32.0 min, and the polydispersity index Mw/Mn amounted to 1.524, indicating that the polysaccharides were widely distributed. The differential RI signal peak was observed within the range of 31.0 to 31.5 min, and the weight average molecular weight was 188.071 kDa, classified as a high-molecular-weight sample (Figure 1B). Further study showed that the crude polysaccharides of Ganoderma lucidum were composed of fucose, rhamnose, arabinose, galactose, glucose, xylose, mannose, galacturonic acid, and glucuronic acid, and their molar mass ratio was 3.76:6.19:2.50:14.03:53.16:4.67:9.77:2.85:3.07, indicating that GLPs were acidic heteropolysaccharides, and glucose and galactose were the main monosaccharides, which was inconsistent with previous research reports [23]. This may be due to different varieties, growth conditions, and extraction methods. The content of glucose was the highest, accounting for 54.03%, which was consistent with previous research reports [24].

### 3.2. Effects of GLPs on Liver Index and Serum Transaminase Levels

Liver indices, ALT, and AST are significant criteria for evaluating liver injury [25]. In comparison with the normal control group, the liver index of the model group, shown in Figure 2B, was notably increased (*p* < 0.01); however, in contrast to the model group, the GLP-L and GLP-M groups demonstrated a marked reduction in the liver index (*p* < 0.05). When contrasted with the normal control group, the ALT activity and AST activity in the model group were significantly elevated (Figure 2C,D), suggesting the successful establishment of the APAP liver injury model. Within this context, compared with the model group, the activities of ALT and AST in the GLP-H group were significantly decreased by 2.74 times and 2.14 times, respectively, while the values in the GLP-L and GLP-M groups were very close to those seen in the positive control group (Figure 2C,D). Thus, the intake of GLPs can reduce APAP-induced liver injury by suppressing serum transaminase activity and the liver index.

### 3.3. Effects of GLPs on Liver Histopathological Changes and Apoptosis

**Figure 3:**
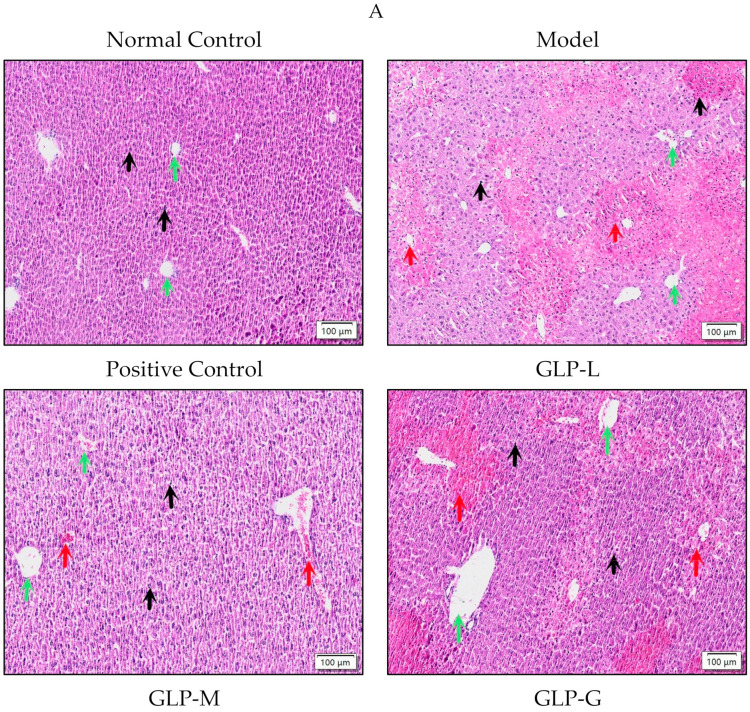

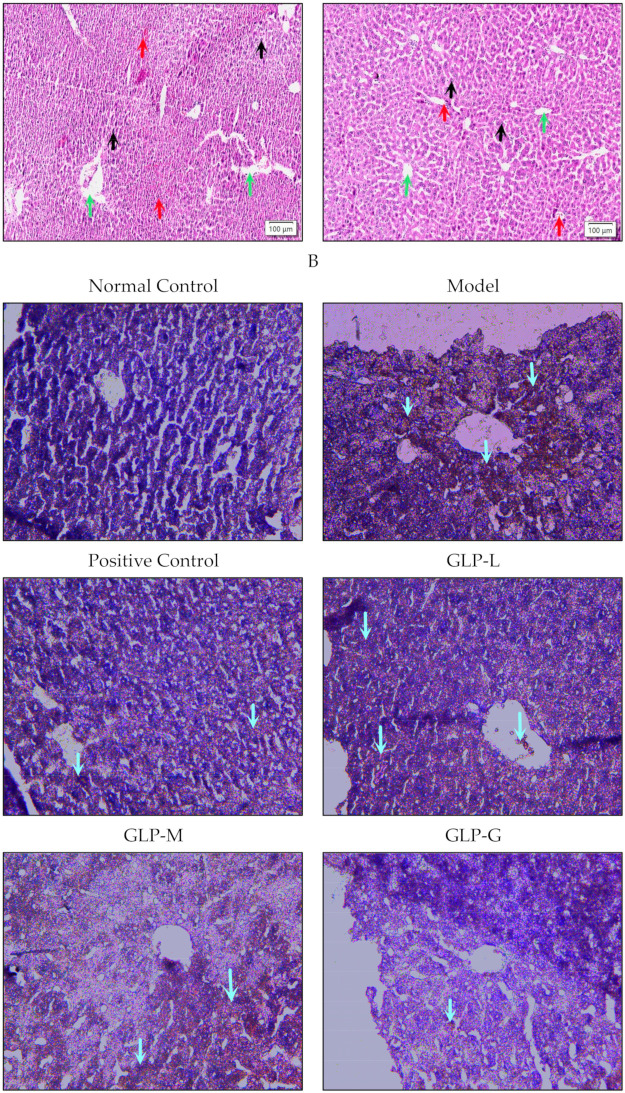

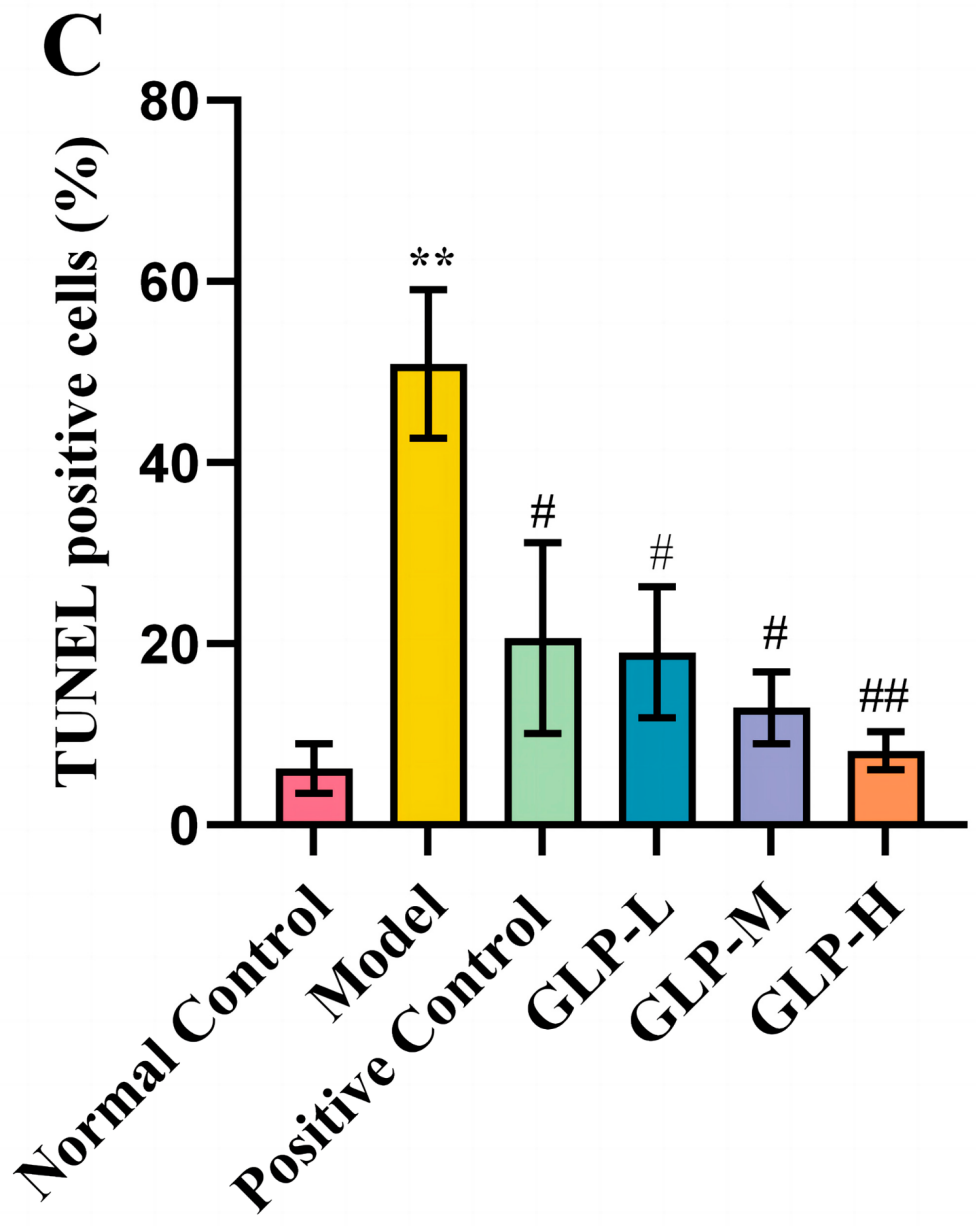
(**A**) HE staining image; (**B**,**C**) TUNEL staining was utilized to observe the apoptosis of liver tissue cells in each group *(n* = 6). The magnification in H&E was ×20, and scale bars = 100 μm; compared with the normal control group, ** *p* < 0.01; compared with the model, # *p* < 0.05, ## *p* < 0.01. The green arrow represents the liver lobules, the black arrow represents the nucleus, the red arrow represents inflammatory cells, and the blue arrow represents cell apoptosis.

In the blank control group, the hepatocyte nucleus was intact, with a clear edge, and no obvious abnormality was detected. The liver tissue of the model group (Figure 3A) exhibited obvious necrosis of hepatic lobules (green arrow), the infiltration of inflammatory cells (red arrow), and the dissolution of liver nuclei (black arrow). After treatment with bifendate and GLPs, these changes caused by APAP were improved. In the GLP-H group, the liver tissue structure was normal, the morphology of hepatocytes was regular, and inflammatory cell infiltration and nucleolysis were significantly reduced, which further confirmed that GLPs alleviated APAP-induced liver injury. TUNEL staining (Figure 3B,C) showed that compared to the blank control group, the number of brown positive cells (blue arrow) in the APAP group increased markedly (*p* < 0.01), which led to the death of hepatocytes. However, bifendate and GLPs could effectively reduce cell apoptosis. Compared with the model group, the number of APAP-induced positive brown cells in the GLP-H group was significantly reduced (*p* < 0.01), indicating that GLPs inhibit APAP-induced apoptosis in liver tissue.

### 3.4. Effects of GLPs on Oxidative Stress Induced by APAP

**Figure 4:**
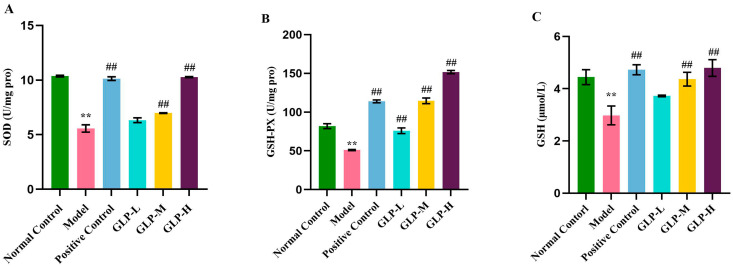

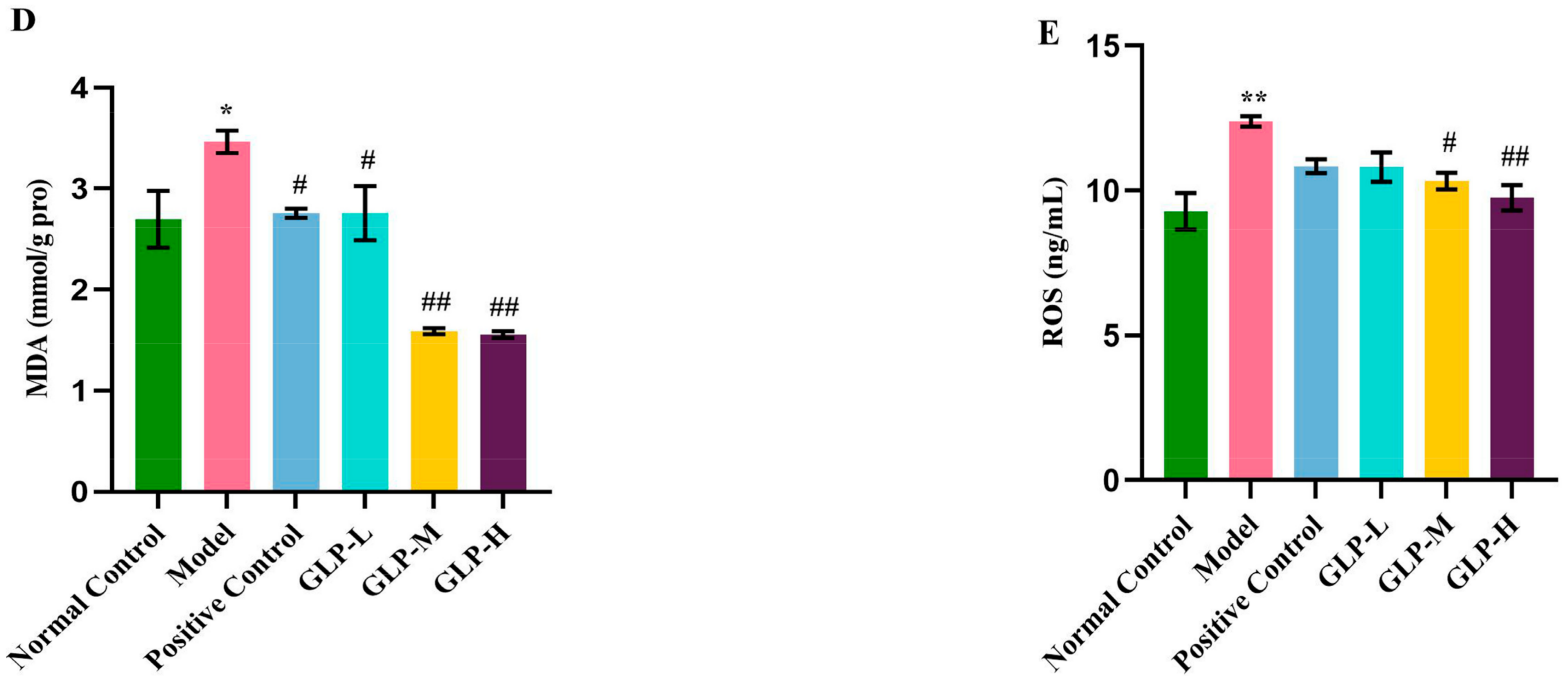
GLPs improve liver antioxidant status: (**A**) SOD activity; (**B**) GSH-Px activity; (**C**) GSH level; (**D**) MDA level; (**E**) ROS level (*n* = 6). The results are expressed as mean ± standard error; compared with the normal control group, * *p* < 0.05, ** *p* < 0.01; compared with the model, # *p* < 0.05, ## *p* < 0.01. Normal control: blank control group, model: model group, positive control: positive group, GLP-L: *Ganoderma lucidum* polysaccharide low-dose group, GLP-M: *Ganoderma lucidum* polysaccharide medium-dose group, GLP-H: *Ganoderma lucidum* polysaccharide high-dose group).

As shown in Figure 4A–E, in comparison with the normal control group, the activities of SOD, GSH-Px, and GSH in the model group were noticeably decreased (*p* < 0.01), and the contents of MDA and ROS were significantly increased (*p* < 0.05), indicating that the oxidative stress induced by APAP was enhanced. In contrast to the model group, bifendate and GLP treatment were able to enhance the activities of SOD, GSH-Px, and GSH, and suppress the production of MDA and ROS. GLPs alleviated oxidative stress in a dose-dependent manner. In particular, in the GLP-H group, the activities of SOD, GSH-Px, and GSH were considerably increased by 1.84, 2.97, and 1.61 times (*p* < 0.01), respectively, and the contents of MDA and ROS were prominently decreased by 2.22 and 1.27 times (*p* < 0.01), respectively. These results imply that GLPs can alleviate the oxidative stress induced by APAP in mice.

### 3.5. Effect of GLPs on mRNA and Protein Expression Related to Nrf2 Pathway in Liver

**Figure 5:**
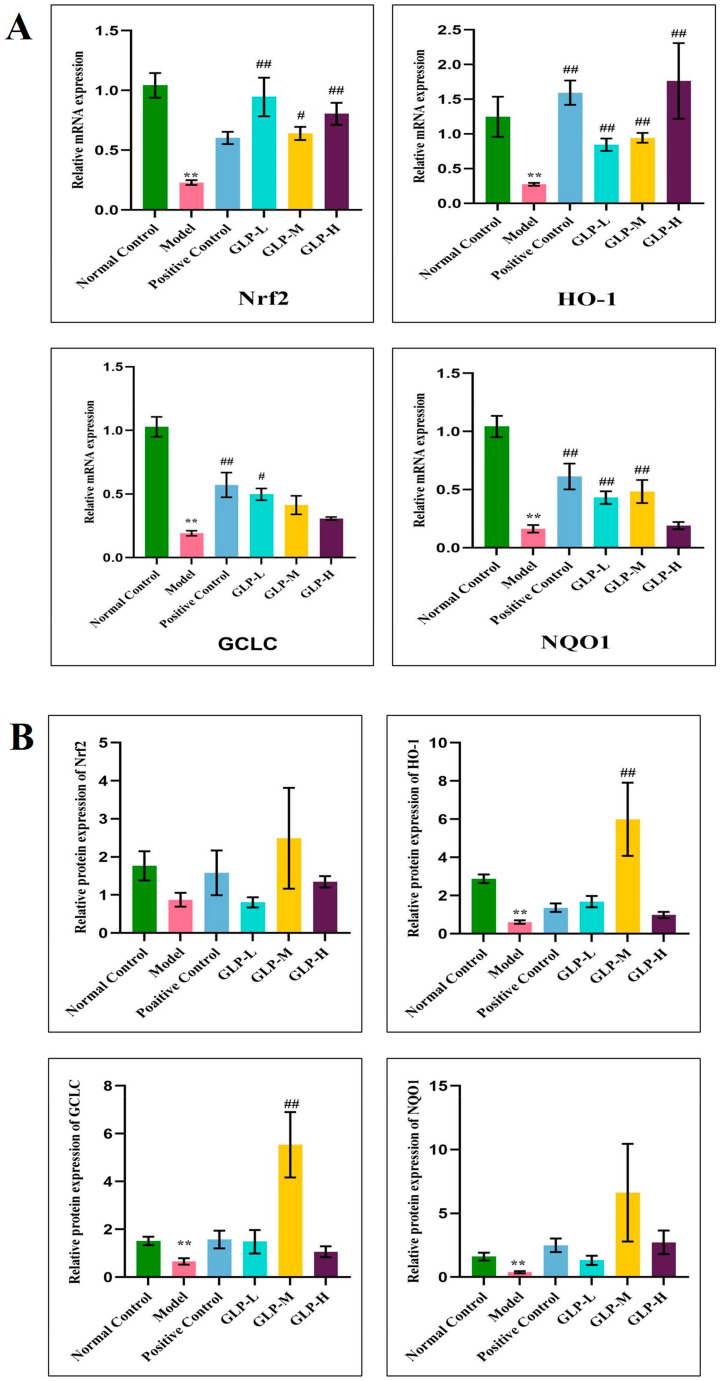

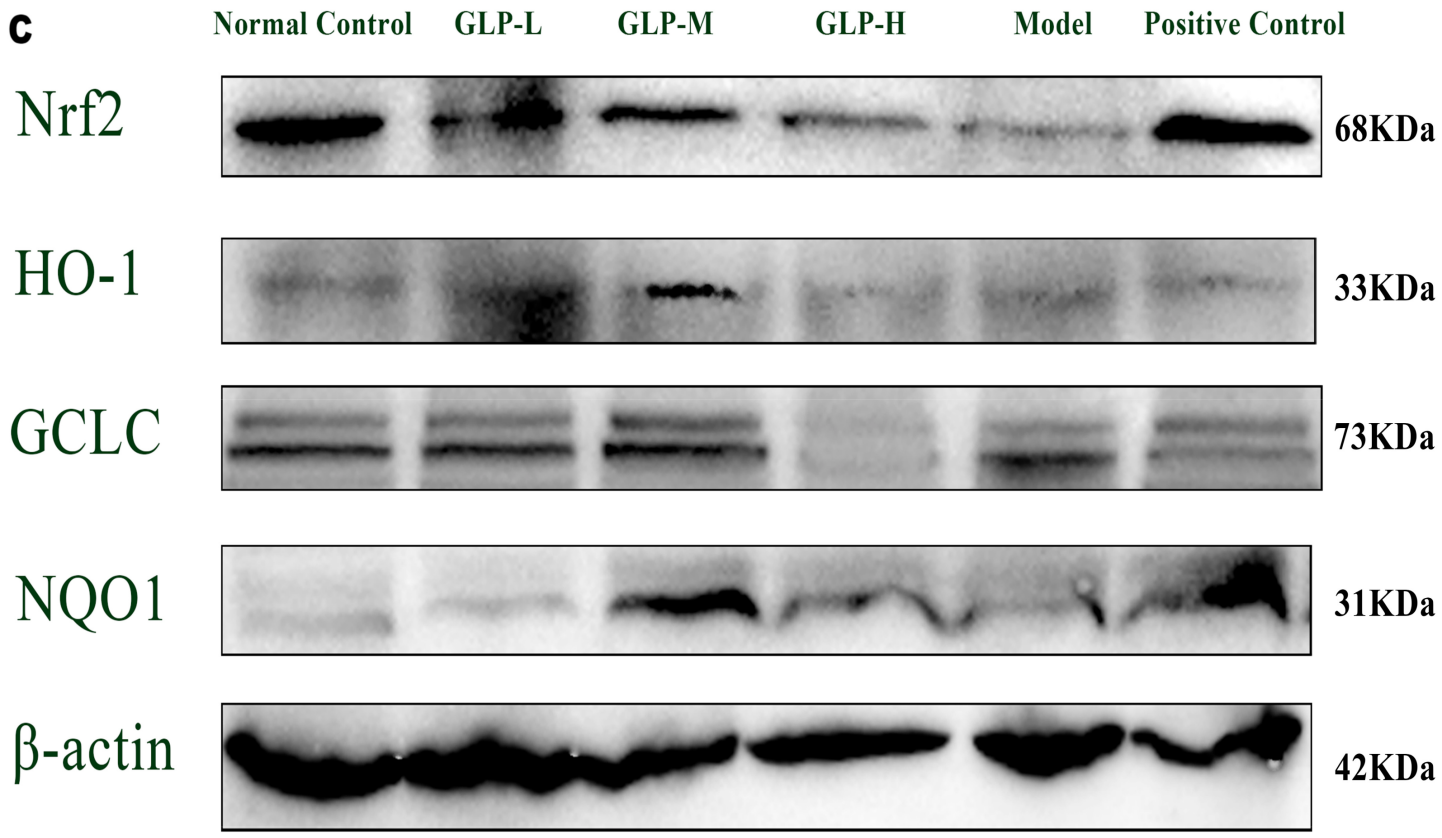
Effect of GLP treatment on the expression of Nrf2 pathway-related genes and proteins: (**A**) Nrf2, HO-1, GCLC, and NQO1 mRNA expression levels in liver tissue; (**B**) quantitative analysis of protein expression (*n* = 6); (**C**) protein imprinting analysis; compared with the normal group, ** *p* < 0.01; compared with the model, # *p* < 0.05, ## *p* < 0.01. Normal control: blank control group, model: model group, positive control: positive group, GLP-L: *Ganoderma lucidum* polysaccharide low-dose group, GLP-M: *Ganoderma lucidum* polysaccharide medium-dose group, GLP-H: *Ganoderma lucidum* polysaccharide high-dose group.

With the aim of further researching the antioxidant mechanism of GLPs in living organisms, the expressions of genes and proteins of Nrf2, HO-1, GCLC, and NQO1 were determined. Compared with the normal control group (Figure 5A), the mRNA levels of Nrf2, HO-1, GCLC, and NQO1 in the model group were significantly reduced (*p* < 0.01). In comparison with the model group, the treatment with GLPs and bifendate reversed the decrease in the expression of genes related to the APAP-induced Nrf2 signaling pathway. In the GLP-H group, the expression of Nrf2 and HO-1 genes was considerably increased, by 3.51 and 3.31 times, respectively (*p* < 0.01), and they were higher than those in the bifendate group. These results imply that GLPs protect the liver from oxidative stress injury by activating the Nrf2 signaling pathway.

In comparison with the normal control group (Figure 5B), the protein expression of HO-1, GCLC, and NQO1 in the model group was significantly decreased (*p* < 0.01), while the protein expression of Nrf2 was decreased, though the difference was not significant. In contrast with the model group, the expression levels of Nrf2, HO-1, GCLC, and NQO1 in biphenyl diester and GLP-L, GLP-M, and GLP-H groups were increased, which was consistent with the mRNA expression magnitude. In the GLP-M group, the expression of Nrf2 and NQO1 protein increased by 2.86 and 17.42 times, respectively, and the presentation of HO-1 and GCLC protein increased by 2.23 and 8.39 times, respectively *p* < 0.01). Notably, there was no significant difference in the protein expression levels of Nrf2, HO-1, GCLC, and NQO1 between the GLP-H, GLP-L, and biphenyl diester groups. These outcomes suggest that GLPs safeguard the liver against oxidative stress by activating the Nrf2 signaling pathway.

## 4. Discussion

Liver injury due to APAP has become a crucial aspect of drug-induced acute liver injury (ALI). Preventing liver toxicity and ensuring the safe use of APAP are highly urgent tasks. At the same time, there is an especially pressing need to develop new drugs to prevent and mitigate ALI brought about by APAP. The APAP-induced ALI mouse model is widely employed to research the role of plants or other therapeutic substances in medication-induced liver damage [26]. Thus, in this research, we utilized this model to determine if taking GLPs can enhance APAP-induced hepatotoxicity.

GLP pretreatment can indeed effectively alleviate APAP-induced liver injury, as evidenced by histomorphological evaluation and biochemical data involving AST and ALT. The study results indicated that APAP notably raised the serum ALT and AST levels in mice, signifying the occurrence of liver injury in the mice. Moreover, histopathological examination also detected central necrosis of hepatic lobules, inflammatory cell infiltration, and nucleolysis, which were further validated by these pathological changes. When GLPs were administered for 7 successive days prior to the injection of toxic doses of APAP, the amounts of ALT and AST in the serum of ALI mice were significantly reduced, especially at 500 mg/kg of GLP, and the therapeutic effect was much higher than that of biphenyl diester and previous research findings [27,28]. The histological inspection of liver tissue sections from ALI mice further validated this protective impact, revealing that the pretreatment with GLPs lessened nuclear deformation and inflammatory cell infiltration. These results collectively confirm that GLPs can prevent APAP-induced liver injury.

Oxidative stress is regarded as the primary mechanism of APAP-induced liver injury [29]. Excessive NAPQI generated by excessive APAP will result in the formation of protein adducts and the generation of ROS. The excessive production of ROS will restrain or deplete the endogenous antioxidant defense, causing oxidative stress, lipid peroxidation, mitochondrial dysfunction, and damage to hepatocytes [30]. Therefore, it is very important to evaluate the level of antioxidant enzymes in the liver for the evaluation of liver injury caused by APAP. SOD and GSH-Px are typical antioxidant enzymes within the liver, while GSH is the most crucial non-enzymatic antioxidant in the organism, and MDA is an important indicator of oxidative stress and lipid peroxidation in the body [31]. Previous investigations have demonstrated that plant polysaccharides can improve the activity and expression of antioxidant enzymes in APAP liver injury mice and inhibit the production of MDA. Phellinus igniarius mycelium polysaccharides can enhance the non-enzymatic and enzymatic antioxidant activities in the liver of APAP-induced acute liver injury mice and reduce the accumulation of ROS and the formation of lipid peroxidation [32]. The polysaccharides of Broussonetia papyrifera leaves can enhance the activities of GSH, GSH-Px, and SOD in the liver of APAP-induced liver injury mice and restore the liver MDA level to normal [33]. These results imply that plant polysaccharides boost the non-enzymatic and enzymatic antioxidant defenses in mice. Comparably, in the results of this study, in contrast to the model group, the intake of GLPs markedly increased the activities of GSH, SOD, and GSH-Px and successfully reduced the contents of ROS and MDA in the liver, presenting a similar effect to the abovementioned polysaccharides. In particular, the therapeutic effect of GLPs in the high-dose group was better than that in the bifendate group, and even better than the abovementioned plant polysaccharides. In addition, the production of ROS can also cause DNA breakage and hepatocyte apoptosis [34]. In this study, TUNEL staining showed that the number of apoptotic hepatocytes in the model group was significantly higher than that in the normal control group, and GLPs could slow down the positive staining of apoptotic cells. These data suggest that the hepatoprotective effect of GLPs may be through the inhibition of oxidative stress and apoptosis.

Nrf2 is a crucial transcription factor within the antioxidant defense system, and it regulates the expression of downstream antioxidant genes through its association with the CIS enhancer sequence ARE [35]. Research has revealed that Nrf2 knockout mice are more susceptible to APAP hepatotoxicity compared to wild-type mice [36]. Platanus orientalis [37], kaempferol [38], and urolithin A [39] can prevent APAP-induced hepatotoxicity by activating the Nrf2 signaling pathway. Hence, the activation of the Nrf2 signaling pathway has come to the fore as a potential treatment modality for APAP-induced liver injury. The findings of this study demonstrated that, in comparison with the model group, GLPs elevated the expression levels of Nrf2 and its downstream HO-1, GCLC, NQO1 protein, and mRNA in APAP-induced liver injury mice, which was concordant with the effect of Leonurus Leonuri in previous research [40]. The results clearly demonstrate that GLPs can regulate the Nrf2 signaling pathway and suppress oxidative stress to improve APAP-induced liver injury.

## 5. Conclusions

In conclusion, for the first time, this research delved into the protective effects of GLPs on APAP-induced liver injury in mice and their possible mechanism from the perspective of oxidative stress. The findings demonstrated that their liver-protective effect was closely associated with alleviating oxidative stress and inhibiting cell apoptosis. GLPs notably reduced the levels of serum ALT and AST, restored the activity of antioxidant enzymes in a dose-dependent manner, activated the Nrf2 signaling pathway, enhanced the antioxidant capacity, and mitigated oxidative stress injury. Additionally, GLPs could effectively inhibit APAP-induced apoptosis. Thus, it is recommended that GLPs be considered as a potential therapeutic agent for preventing or treating APAP-induced liver injury.

## Figures and Tables

**Table 1:** Antibody list.

Antibody Name	Dilution Ratio	Item Number
Nrf2	1:1000	A21176
HO-1	1:10,000	A19062
GCLC	1:3000	A1038
NQO1	1:1000	A1518
β-actin	1:50,000	AC026
IgG(H + L)	1:5000	A0208

**Table 2:** Primer sequence of qRT-PCR.

Target Gene	Primer	Sequence(5′–3′)
Nrf2	Forward	CGAGATATACGCAGGAGAGGTAAGA
	Reverse	GCTCGACAATGTTCTCCAGCTT
HO-1	Forward	TGCAGGTGATGCTGACAGAGG
	Reverse	GGGATGAGCTAGTGCTGATCTGG
GCLC	Forward	CAGTCAAGGACCGGCACAAG
	Reverse	CAAGAACATCGCCTCCATTCAG
NQO1	Forward	CAGCCAATCAGCGTTCGGTA
	Reverse	CTTCATGGCGTAGTTGAATGATGTC
β-actin	Forward	GGCTGTATTCCCCTCCATCG
	Reverse	CCAGTTGGTAACAATGCCATGT

## Data Availability

The datasets generated during and/or analyzed during the current study are either shown in the manuscript or are available from the corresponding author upon reasonable request.
